# Association between dietary live microbe intake and Life's Essential 8 in US adults: a cross-sectional study of NHANES 2005–2018

**DOI:** 10.3389/fnut.2024.1340028

**Published:** 2024-02-29

**Authors:** Lin Wang, Sutong Wang, Yongcheng Wang, Shuli Zong, Zhaoyu Li, Yuehua Jiang, Xiao Li

**Affiliations:** ^1^First Clinical Medical College, Shandong University of Traditional Chinese Medicine, Jinan, China; ^2^Department of Cardiovascular Diseases, Affiliated Hospital of Shandong University of Traditional Chinese Medicine, Jinan, China; ^3^College of Traditional Chinese Medicine, Shandong University of Traditional Chinese Medicine, Jinan, China; ^4^Central Laboratory, Affiliated Hospital of Shandong University of Traditional Chinese Medicine, Jinan, China

**Keywords:** cross-sectional study, dietary live microbe, Life's Essential 8, cardiovascular health, NHANES

## Abstract

**Background:**

Assessing the impact of dietary live microbe intake on health outcomes has gained increasing interest. This study aimed to elucidate the relationship between dietary live microbe intake and Life's Essential 8 (LE8) scores, a metric for cardiovascular health (CVH), in the U.S. adult population.

**Methods:**

We analyzed data from 10,531 adult participants of the National Health and Nutrition Examination Survey (NHANES) spanning 2005–2018. Participants were stratified into low, medium, and high intake groups of dietary live microbe based on Marco's classification system. We employed weighted logistic and linear regression analyses, along with subgroup, interaction effect, and sensitivity analyses. Additionally, Restricted Cubic Splines (RCS) were used to explore the dose-response relationship between food intake and CVH in different groups.

**Results:**

Compared to the low live microbe intake group, the medium and high live microbe intake groups had significantly higher LE8, with β coefficients of 2.75 (95% CI: 3.89–5.65) and 3.89 (95% CI: 6.05–8.11) respectively. Additionally, moderate and high groups significantly reduced the risk of high cardiovascular health risk, defined as an LE8 score below 50, with odds ratios (OR) of 0.73 and 0.65 respectively. Subgroup analysis and sensitivity analysis proved the stability of the results. In the low intake group, food intake shows a linear negative correlation with LE8, whereas in the high intake group, it exhibits a linear positive correlation. In contrast, in the moderate live microbe intake group, the relationship between food intake and LE8 presents a distinct inverted “U” shape.

**Conclusion:**

This study highlights the potential benefits of medium to high dietary intake of live microbe in improving LE8 scores and CVH in adults. These findings advocate for the inclusion of live microbes in dietary recommendations, suggesting their key role in CVH enhancement.

## 1 Introduction

While advancements in food hygiene and environmental sanitation have markedly elevated public health standards, the concomitant reduction in microbial exposure may elicit unforeseen detrimental effects (1). The contemporary scientific milieu has witnessed a burgeoning recognition of the salutary potential harbored by microbial ingestion for human health (2). The “Old Friends Hypothesis” provides a compelling narrative on the integral role of microbes, positing that exposure to symbiotic or innocuous microbes present in our diet serves as a crucial conduit for beneficial microbial stimulation of the immune system (3). The ingestion of live, benign microbes, as part of our daily dietary intake, facilitates their transit to the gut where they seamlessly assimilate with the resident microbial consortium, thereby augmenting gut functionality, orchestrating immune system modulation, and ultimately attenuating susceptibility to chronic ailments (1).

Cardiovascular diseases (CVD) remain a leading cause of death in developed countries and a major global health issue, despite advanced lipid-lowering drugs. Their high incidence rates continue to heavily impact society and economic development (4, 5). A well-known factor contributing to cardiovascular health (CVH) is dietary patterns (6). The gut microbiota can convert commonly consumed nutrients in food into metabolites, some of which are closely related to cardiovascular diseases (7). The gut microbiota has been implicated in cardiovascular diseases in epidemiological studies and animal experiments in the past (8, 9). To enhance the prevention of CVD and consequently reduce their incidence, the American Heart Association (AHA) introduced a novel concept of cardiovascular health in 2010, termed Life's Simple 7 (LS7). This paradigm shift transformed the approach to disease management from mere treatment to fostering and safeguarding the health of individuals and communities throughout their lifespan (10). In 2022, following the optimization of the LS7 scoring scale, the AHA unveiled a new CVH score, Life's Essential 8 (LE8). The LE8 is comprised of two primary components, covering four health behaviors (diet, physical activity (PA), nicotine exposure, and sleep health) as well as four health factors [body mass index (BMI), blood pressure (BP), blood lipids and blood glucose] (11).

Although several studies have explored the connection between dietary live microbes and health, their impact on LE8 remains unclear (12–14). A nationally representative sample from the National Health and Nutrition Examination Survey (NHANES) was used in this study to define and estimate the intake of dietary live microbes. We then assess the relationship between dietary live microbe intake and the LE8 score (15). Given the intricate interplay between dietary live microbe intake and CVD, our study pioneers the exploration of the potential linkage between dietary live microbe intake and the LE8 score.

## 2 Method

### 2.1 Study design and population

The NHANES database employs a complex, multistage probability sampling design to reflect the nutritional and health status of the U.S. population. This study was approved by the National Center for Health Statistics (NCHS) Institutional Review Board, and all participants provided written informed consent. Notably, the NHANES database is publicly accessible and does not require additional ethical or administrative approval for use.

**Figure 1:**
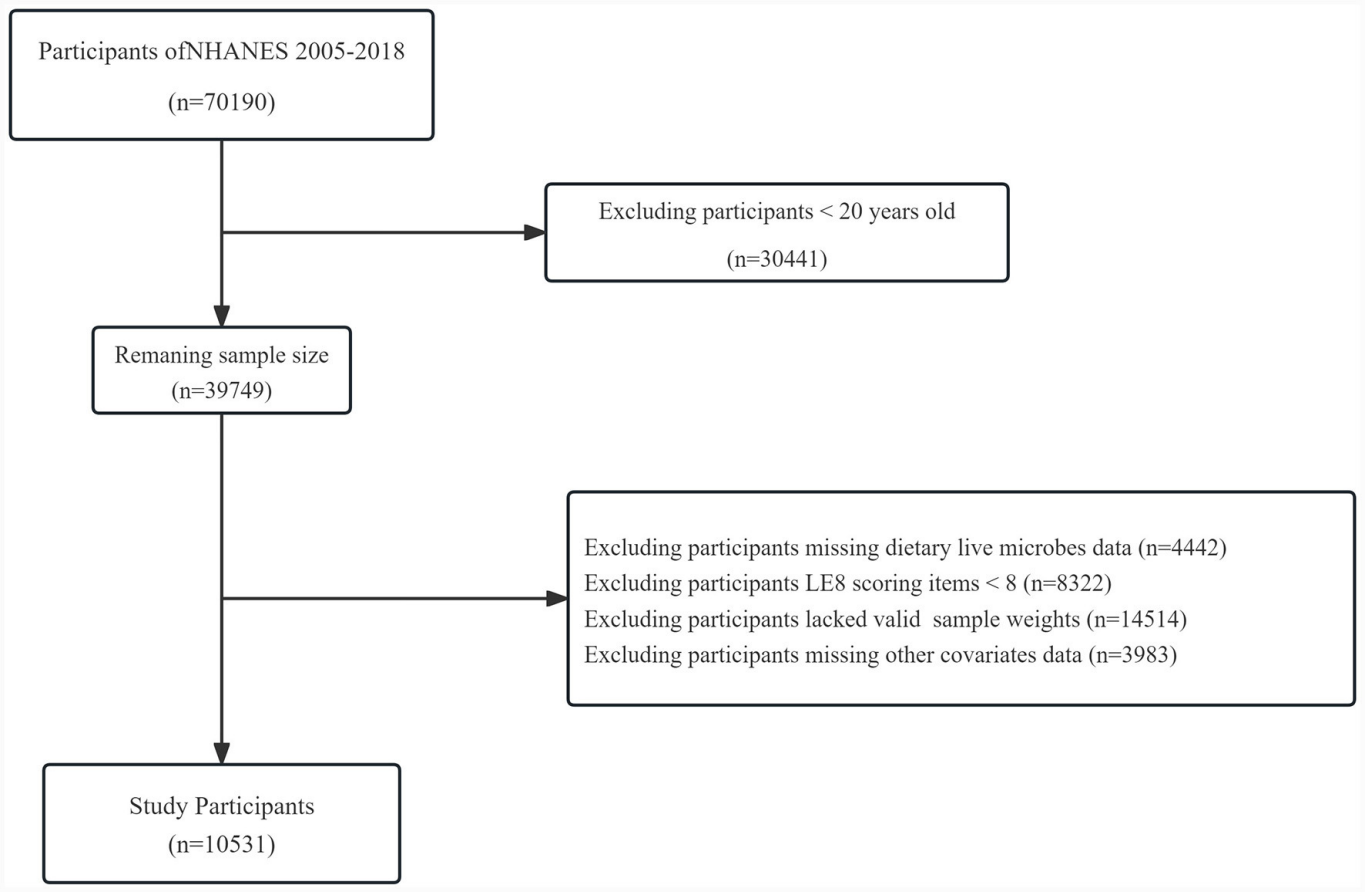
Flow chart of the study.

Our study meticulously adheres to the STROBE guidelines (16). Sleep health is a key factor in assessing the LE8, and applicable data in NHANES are only available after 2005. Therefore, our study incorporated data from a total of seven survey cycles between 2005 and 2018. A total of 70,190 individuals participated in the survey. Among them, 30,441 were under the age of 20, 4,422 lacked information on dietary live microbe intake, 8,322 lacked information on the LE8, 14,514 lacked valid sample weights, and 3,983 lacked information on other covariates. As shown in Figure 1, after screening, a total of 10,531 participants were finally included in the study.

### 2.2 Dietary intakes and live microbial category

In the NHANES dataset, the dietary intake of participants during two distinct 24-h periods is thoroughly recorded through face-to-face interviews and telephone follow-ups. The NCHS uses dietary nutritional data from the United States Department of Agriculture (USDA) to accurately assess the energy and nutritional content of each food and beverage. The estimated quantity of live microbes (per gram) in 9,388 foods across 48 subgroups in the NHANES database was determined by a team of four experts, including Marco. During these assessments, the experts relied on reported values in professional literature and authoritative reviews, or made inferences based on the known effects of food processing on microbial vitality. Foods were categorized into low (Lo; < 10^4^ CFUs/g), medium (Med; 10^4^-10^7^ CFU/g), and high (Hi; >10^7^ CFU/g) classes based on their live microbe content. Any discordance encountered during the assessment of live microbe content was resolved through external consultations and discussions.

### 2.3 Assessment of LE8

Each of the eight indicators is scored according to publicly available official calculation methodologies, with the specific calculation methods detailed in the Supplementary Table 1. Within the eight CVH indicators, each indicator is scored on a scale ranging from 0 to 100. The overall LE8 score is derived from the unweighted average of these indicators. Based on the final score, CVH is categorized into three groups: low (0–49 points), medium (50–79 points), and high (80–100 points) (17).

The dietary indicator was assessed using the 2015 Healthy Eating Index (HEI) (18), and our study included participants with 2 days of dietary data (those with only 1 day of dietary data were excluded). Subsequently, dietary information was combined with data from the USDA to calculate the HEI (19), details are provided in the Supplementary Table 2. Self-reported survey questionnaires were used to collect information on participants' PA, smoking, sleep, history of diabetes mellitus (DM), and medication use. Height, weight, and BP were measured in mobile examination centers, and BMI was calculated. Blood samples were collected and sent to a central laboratory for testing of blood lipids, blood glucose, and glycated hemoglobin. Non-high-density lipoprotein cholesterol was calculated as total cholesterol minus high-density lipoprotein cholesterol.

### 2.4 Covariates and other variables

In light of prior studies and clinical experiences (12, 20), confounding factors that could influence the relationship between dietary microbes and CVH were taken into consideration. These factors included age, gender and race/ethnicity (non-Hispanic White, non-Hispanic Black, Mexican American, other Hispanic, and other race—including multi-racial). Educational level was also considered, categorized as less than high school, high school graduate/GED or equivalent, and college graduate or above. Marital status was classified into married/living with partner, never married, and widowed/divorced/separated. Economic status was assessed using the poverty income ratio (PIR) with categories of < 1.30, 1.3–3.49, and 3.50 or higher. Health insurance status was noted as either Yes or No. Alcohol consumption included never drinking, former drinking, mild drinking, moderate drinking and heavy drinking. Obesity status includes normal weight, overweight and obesity. Medical history encompassed a history of CVD (Yes, No), DM (Yes, No), hypertension (HTN; Yes, No), and hyperlipidemia (HLD; Yes, No). The details of these variable assessments can be found in Supplementary Table 3. Additionally, we assessed the participants' average daily intake of energy, protein, carbohydrates, dietary fiber, and fat.

### 2.5 Statistical method

To ensure national representation, this study considered sample weights in all analyses. Participants were grouped into Low, Medium, and High dietary live microbe categories, where each group corresponds to the consumption level of foods within microbial content ranges, with the Low group consuming mostly low microbe-content foods, the Medium group consuming a balance excluding high microbe-content foods, and the High group predominantly eating foods rich in live microbes. We conducted one-way ANOVA (for continuous variables) and Chi-square tests (for categorical variables) to assess the baseline characteristics of participants among different groups. Continuous variables are represented by mean ± standard deviation, while categorical variables are expressed as the number of cases (*n*) and weighted percentages (%).

Weighted univariate and multivariate linear regression models are used to explore the relationship between dietary live microbes and LE8. Additionally, health behaviors and health factors were explored separately. To delve deeper into the connection between them, we consider a LE8 score below 50 as an indicator of high cardiovascular health risk (HCVHR). Weighted univariate and multivariate logistic regression models are used to explore the relationship between live microbes intake and HCVHR. In addition to the univariate model, we also developed three multivariate regression models. Model 1 was adjusted for age, gender, race/ethnicity, and education level. Model 2, building upon model 1, further incorporated adjustments for PIR, health insurance, marital status, and alcohol consumption. Finally, Model 3, expanding upon Model 2, additionally adjusted for dietary components including energy, protein, carbohydrate, fat, and fiber intake.

We also conducted subgroup analyses and interaction analyses based on age, gender, race/ethnicity, education level, PIR, health insurance, and marital status. Moreover, in each group with low, medium, and high live microbes intake, we employed the restricted cubic spline (RCS) analysis method to investigate the relationship between the quantity of food consumed (grams) and LE8. Finally, for the purpose of sensitivity analysis, we excluded individuals with a history of CVD, DM, HLD, and HTN. Statistical analyses were conducted using R software, version 4.3.1. A two-tailed *P*-value of < 0.05 was considered to indicate statistical significance.

## 3 Result

### 3.1 Characteristics of participants across different dietary live microbe intake groups

Participants were categorized into three groups based on their intake levels of dietary live microbes: low, medium, and high. As shown in Table 1, the average age of the population included in the study is 47.59 years, with a slightly higher proportion of females than males. The predominant ethnicity is non-Hispanic White, and the majority of participants have a college education or higher. Most participants have health insurance, a habit of drinking alcohol, and are either married or cohabiting with a partner. In terms of weight status, the majority are obese, with 8.81% of participants suffering from CVD, 13.87% having DM, 37.44% experiencing HTN, and over 70% suffering from HLD. The assessment of CVH shows that 66.34% of the participants are at a moderate level. Among the three groups of participants, there were significant differences in all aspects except for daily carbohydrate intake, prevalence of HTN, and HLD. We also compiled the baseline characteristics of participants categorized by low, medium, and high levels of CVH, as shown in Supplementary Table 4.

**Table 1:** The clinical characteristics of the study population according to the different dietary live microbes.

**Characteristic**	**Total (*n* = 10,531)**	**Low dietary live microbe group (*n* = 3,719)**	**Medium dietary live microbe group (*n* = 4,339)**	**High dietary live microbe group (*n* = 2,473)**	***P*-value**
Age (years)	47.59 ± 0.30	45.52 ± 0.40	49.22 ± 0.40	47.63 ± 0.46	< 0.0001
**Age group (%)**					< 0.0001
20–40	3,379 (35.49)	1,294 (40.14)	1,258 (31.97)	827 (35.17)	
40–60	3,567 (38.36)	1,256 (37.88)	1,457 (38.79)	854 (38.29)	
≥60	3,585 (26.15)	1,169 (21.97)	1,624 (29.25)	792 (26.54)	
**Gender (%)**					< 0.0001
Female	5,413 (51.80)	1,803 (47.40)	2,227 (52.07)	1,383 (56.30)	
Male	5,118 (48.20)	1,916 (52.60)	2,112 (47.93)	1,090 (43.70)	
**Race/ethnicity (%)**					< 0.0001
Non-Hispanic White	4,971 (71.16)	1,545 (65.03)	2,019 (70.03)	1,407 (79.50)	
Non-Hispanic Black	2,052 (10.28)	1,025 (15.98)	744 (9.24)	283 (5.38)	
Mexican American	1,547 (7.36)	468 (7.01)	778 (9.27)	301 (5.15)	
Other Hispanic	959 (4.78)	326 (5.10)	401 (4.82)	232 (4.36)	
Other race	1,002 (6.42)	355 (6.88)	397 (6.65)	250 (5.61)	
**Education level (%)**					< 0.0001
Less than high school	2,255 (13.97)	943 (18.80)	970 (14.18)	342 (8.32)	
High School Graduate/GED or Equivalent	2,401 (22.80)	967 (26.89)	973 (22.28)	461 (18.98)	
College Graduate or above	5,875 (63.23)	1,809 (54.31)	2,396 (63.54)	1,670 (72.70)	
**PIR (%)**					< 0.0001
< 1.30	3,031 (19.21)	1,306 (26.49)	1,190 (17.20)	535 (13.90)	
1.3–3.49	4,067 (36.48)	1,528 (40.10)	1,677 (36.84)	862 (31.99)	
≥3.50	3,433 (44.31)	885 (33.41)	1,472 (45.97)	1,076 (54.10)	
**Health insurance (%)**					< 0.0001
Yes	8,447 (84.00)	2,856 (78.73)	3,496 (84.74)	2,095 (88.81)	
No	2,084 (16.00)	863 (21.27)	843 (15.26)	378 (11.19)	
**Marital status (%)**					< 0.0001
Married/living with partner	6,444 (64.98)	2,077 (59.00)	2,778 (67.32)	1,589 (68.41)	
Never married	1,828 (17.38)	736 (20.43)	671 (15.61)	421 (16.43)	
Widowed/divorced/separated	2,259 (17.64)	906 (20.58)	890 (17.08)	463 (15.16)	
**Alcohol consumption (%)**					< 0.0001
Never	1,341 (10.20)	497 (11.04)	571 (10.80)	273 (8.45)	
Former	1,751 (13.39)	710 (15.11)	715 (13.59)	326 (11.24)	
Mild	3,796 (38.76)	1,177 (34.20)	1,617 (40.09)	1,002 (41.96)	
Moderate	1,636 (17.84)	562 (16.91)	642 (16.69)	432 (20.46)	
Heavy	2,007 (19.80)	773 (22.73)	794 (18.83)	440 (17.90)	
LE8	69.03 ± 0.29	65.11 ± 0.38	69.88 ± 0.34	72.19 ± 0.42	< 0.0001
Energy intake (kcal/day)	2,101.73 ± 10.14	2,034.48 ± 21.25	2,103.79 ± 15.57	2,173.35 ± 20.16	< 0.0001
Protein intake (g/day)	82.77 ± 0.44	77.80 ± 0.80	83.19 ± 0.75	87.70 ± 0.83	< 0.0001
Carbohydrate intake (g/day)	250.22 ± 1.37	246.98 ± 2.86	251.61 ± 1.96	251.91 ± 2.80	0.35
Fiber intake (g/day)	17.04 ± 0.16	14.51 ± 0.20	18.14 ± 0.22	18.35 ± 0.25	< 0.0001
Fat intake (g/day)	81.33 ± 0.47	77.50 ± 0.92	81.06 ± 0.74	85.94 ± 0.94	< 0.0001
BMI (kg/m^2^)	28.96 ± 0.11	29.67 ± 0.21	28.86 ± 0.14	28.31 ± 0.15	< 0.0001
**Obesity (%)**					< 0.0001
Normal weight	3,058 (30.41)	1,015 (27.93)	1,261 (30.79)	782 (32.63)	
Over weight	3,487 (33.05)	1,165 (31.14)	1,473 (32.93)	849 (35.34)	
Obesity	3,986 (36.54)	1,539 (40.93)	1,605 (36.28)	842 (32.03)	
**CVD (%)**					0.004
Yes	1,163 (8.81)	457 (9.79)	487 (9.13)	219 (7.28)	
No	9,368 (91.19)	3,262 (90.21)	3,852 (90.87)	2,254 (92.72)	
**DM (%)**					< 0.001
Yes	1,980 (13.87)	706 (14.15)	897 (15.70)	377 (11.05)	
No	8,551 (86.13)	3,013 (85.85)	3,442 (84.30)	2,096 (88.95)	
**Hypertension (%)**					0.05
Yes	4,472 (37.44)	1,637 (38.98)	1,889 (38.05)	946 (34.91)	
No	6,059 (62.56)	2,082 (61.02)	2,450 (61.95)	1,527 (65.09)	
**Hyperlipidemia (%)**					0.07
Yes	7,583 (70.53)	2,692 (71.33)	3,173 (71.47)	1,718 (68.35)	
No	2,948 (29.47)	1,027 (28.67)	1,166 (28.53)	755 (31.65)	
**CVH**^†^ **(%)**					< 0.0001
Low	1,245 (9.41)	593 (13.52)	456 (8.32)	196 (6.35)	
Moderate	7,080 (66.34)	2,614 (71.22)	2,926 (66.37)	1,540 (60.90)	
High	2,206 (24.25)	512 (15.26)	957 (25.31)	737 (32.75)	
**HCVHR**^‡^ **(%)**					< 0.0001
Yes	1,245 (9.41)	593 (13.52)	456 (8.32)	196 (6.35)	
No	9,286 (90.59)	3,126 (86.48)	3,883 (91.68)	2,277 (93.65)	
**Year cycle (%)**					< 0.0001
2005–2010	4,722 (42.75)	1,553 (40.38)	2,193 (49.70)	976 (35.84)	
2010–2018	5,809 (57.25)	2,166 (59.62)	2,146 (50.30)	1,497 (64.16)	

LE8, Life's Essential 8; BMI, body mass index; HEI, healthy eating index; SBP, systolic blood pressure; DBP, diastolic blood pressure; PIR, ratio of family income to poverty; CVD, cardiovascular disease; DM, diabetes mellitus; CVH, cardiovascular health; HCVHR, high cardiovascular health risk.

^†^Low CVH was defined as a LE8 score of 0–49, moderate CVH of 50–79, and high CVH of 80–100.

^‡^HCVHR was defined as participants with LE8 scores < 50.

### 3.2 Association between different dietary live microbe groups and LE8

Table 2 shows the association between different dietary live microbes intake and LE8. In the crude model, compared to the low group (reference group), the medium and high groups showed significantly higher LE8 scores, with estimated values (β) of 4.77 (95% CI: 3.89–5.65) and 7.08 (95% CI: 6.05–8.11), respectively, both reaching statistical significance (*P* < 0.0001). In the multivariate-adjusted models, the association between dietary live microbe groups and LE8 remained significant. In Model 1, the β coefficients for the medium and high groups were estimated at 4.54 (95% CI: 3.76–5.31) and 5.66 (95% CI: 4.65–6.67), respectively. The estimates slightly decreased in Model 2, with the medium group at 3.98 (95% CI: 3.23–4.74) and the high group at 4.94 (95% CI: 3.95–5.92). In the final Model 3, the β coefficients for the medium and high dietary groups further reduced to 2.75 (95% CI: 2.01–3.50) and 3.89 (95% CI: 2.97–4.81), while the associations remained significant (*P* < 0.0001).

**Table 2:** Association between different dietary live microbe group and LE8.

**Outcome**	**Model**	**Low dietary live microbe group β (95% CI)**	**Medium dietary live microbe group β (95% CI)**	**High dietary live microbe group β (95% CI)**	***P* for trend**
LE8	Crude	1.00 (reference)	4.77 (3.89, 5.65)^****^	7.08 (6.05, 8.11)^****^	< 0.0001
	Model 1	1.00 (reference)	4.54 (3.76, 5.31)^****^	5.66 (4.65, 6.67)^****^	< 0.0001
	Model 2	1.00 (reference)	3.98 (3.23, 4.74)^****^	4.94 (3.95, 5.92)^****^	< 0.0001
	Model 3	1.00 (reference)	2.75 (2.01, 3.50)^****^	3.89 (2.97, 4.81)^****^	< 0.0001

Model 1: Adjusted for age (as a continuous variable), gender, race/ethnicity, and education level.

Model 2: Further adjusted for PIR, health insurance, marital status, and alcohol consumption.

Model 3: Further adjusted for energy intake, protein intake, carbohydrate intake, fat intake, and fiber intake.

^****^P value < 0.0001.

As previously mentioned, LE8 primarily comprises two components: health factors and health behaviors. We further explored the relationship between these components and the intake of live microbes. As shown in Supplementary Table 4, across all analysis models, groups with higher intake of live microbes were generally associated with higher scores in health factors and health behaviors, and this association remained significant in the multivariate-adjusted models. It is noteworthy that in the crude model, there was no significant difference between the medium and low dietary live microbe groups.

### 3.3 Association between different dietary live microbe groups and HCVHR

To further explore the relationship between different levels of live microbes intake and the risk of cardiovascular health, we employed logistic regression analysis. The study builds on previous linear regression analyses and defines individuals with an LE8 score below 50 as HCVHR. As shown in Table 3, compared to low group, moderate and high groups are associated with a significantly reduced risk of cardiovascular health. In the crude model, without adjusting for any confounding factors, moderate live microbes intake was associated with a reduced risk of cardiovascular health (OR = 0.58, 95% CI: 0.49–0.69), and the reduction was more significant for high live microbes intake (OR = 0.43, 95% CI: 0.34–0.55), with a *P*-value for the trend < 0.0001. This trend remained consistent after progressively adjusting for confounding factors. In the final model, taking into account a variety of potential confounders, moderate and high groups were still significantly associated with lower cardiovascular health risk. Specifically, the odds ratio for moderate live microbes intake was 0.73 (95% CI: 0.61–0.89), and for high live microbes intake, it was 0.65 (95% CI: 0.50–0.84).

**Table 3:** Association between different dietary live microbe groups and HCVHR.

**Outcome**	**Model**	**Low dietary live microbe group OR (95% CI)**	**Medium dietary live microbe group OR (95% CI)**	**High dietary live microbe group OR (95%CI)**	***P* for trend**
HCVHR	Crude	1.00 (reference)	0.58 (0.49, 0.69)^****^	0.43 (0.34, 0.55)^****^	< 0.0001
	Model 1	1.00 (reference)	0.59 (0.49, 0.71)^****^	0.52 (0.40, 0.67)^****^	< 0.0001
	Model 2	1.00 (reference)	0.64 (0.53, 0.77)^****^	0.58 (0.45, 0.75)^****^	< 0.0001
	Model 3	1.00 (reference)	0.73 (0.61, 0.89)^**^	0.65 (0.50, 0.84)^**^	< 0.0001

Model 1: Adjusted for age (as a continuous variable), gender, race/ethnicity, and education level.

Model 2: Further adjusted for PIR, health insurance, marital status, and alcohol consumption.

Model 3: Further adjusted for energy intake, protein intake, carbohydrate intake, fat intake, and fiber intake.

^**^P value < 0.01.

^****^P value < 0.0001.

### 3.4 Subgroup analysis and interaction analysis

**Figure 2:**
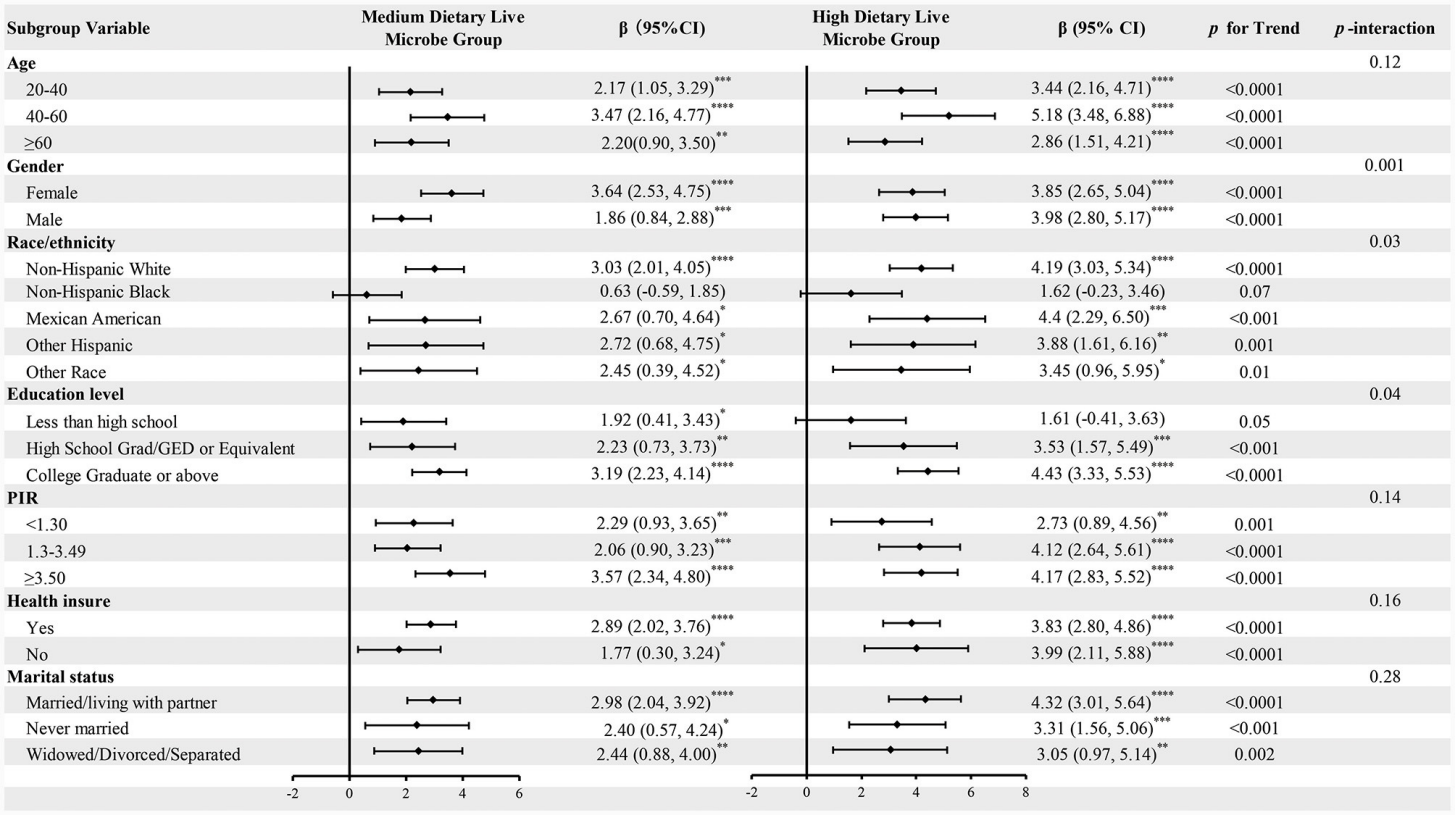
Subgroup analysis for the associations of different dietary live microbe intake and LE8. The model was adjusted for age, gender, race/ethnicity, education level, PIR, health insurance, marital status, alcohol consumption, energy intake, protein intake, carbohydrate intake, fat intake, and fiber intake when they were not the strata variables. *P < 0.05; **P < 0.01; ***P < 0.001; ****P < 0.0001.

To investigate the consistent link between dietary live microbes intake and CVH across various populations, subgroup analyses were conducted. The analysis covered various population subgroups, including age, gender, race, education level, household income ratio, health insurance status, and marital status. As shown in Figure 2, the results indicate a significant positive correlation between the intake of live microbes and LE8, which was validated in the vast majority of subgroups. However, there were some non-significant results in specific subgroups, such as non-Hispanic blacks, suggesting caution when generalizing these findings to a broader population. Trend tests showed that as the intake of live microbes increased (from moderate to high amounts), their positive impact on CVH tended to increase across all subgroups. Interaction analysis revealed that the relationship between dietary live microbe levels and LE8 was significantly influenced by gender, race and education level. The impact of gender was particularly significant (*P* = 0.001), suggesting that men and women might respond differently to the intake of live microbes. Additionally, subgroup analyses on the relationship between dietary live microbes levels and HCVHR were also conducted, with the results largely consistent (Supplementary Figure 1).

### 3.5 Sensitivity analysis

To verify the reliability of results in a healthy population, we also excluded individuals with a history of cardiovascular and related diseases. We conducted five sensitivity analyses, each excluding populations with a history of CVD, DM, HLD, HTN, and any combination of these conditions. Multivariable regression analyses were carried out under the fully adjusted model (Model 3), with the results presented in Table 4. Across the five different sets of exclusion criteria, the positive correlation between moderate to high intake of dietary live microbes and LE8 was consistently significant. Excluding individuals with CVD history (*n* = 9,368), both moderate (β = 2.64, 95% CI: 1.84–3.45, *P* < 0.0001) and high (β = 3.88, 95% CI: 2.90–4.86, *P* < 0.0001) intake groups showed significant positive associations. Excluding DM history (*n* = 8,551), results were similar (Moderate: β = 2.73, High: β = 3.66; both *P* < 0.0001). Excluding HLD history (*n* = 2,948), high intake remained significantly associated (β = 2.50, 95% CI: 1.06–3.95, *P* < 0.0001), while moderate intake showed no significant association. Excluding HTN history (*n* = 6,059), both intake groups showed significant associations (Moderate: β = 2.85, High: β = 3.81; both *P* < 0.0001). Excluding all above conditions (*n* = 2,050), significant associations persisted (Moderate: β = 2.04, High: β = 3.06; both *P* < 0.0001).

**Table 4:** Sensitivity analysis of the association of the different dietary live microbe intake and LE8.

**Outcome**	**Excluded history**	**Sample size (*n*)**	**Low dietary live microbe group β (95% CI)**	**Medium dietary live microbe group β (95% CI)**	**High dietary live microbe group β (95% CI)**	***P* for trend**
LE8	CVD	9,368	1.00 (reference)	2.64 (1.84, 3.45)^****^	3.88 (2.90, 4.86)^****^	< 0.0001
	DM	8,551	1.00 (reference)	2.73 (2.03, 3.43)^****^	3.66 (2.78, 4.53)^****^	< 0.0001
	HLD	2,948	1.00 (reference)	1.10(-0.07, 2.27)	2.50 (1.06, 3.95)^***^	< 0.0001
	HTN	6,059	1.00 (reference)	2.85 (1.98, 3.73)^****^	3.81 (2.75, 4.86)^****^	< 0.0001
	All the above conditions	2,050	1.00 (reference)	2.04 (0.85, 3.22)^***^	3.06 (1.58, 4.54)^****^	< 0.0001

CVD, cardiovascular disease; DM, diabetes mellitus; HLD, hyperlipidemia; HTN, hypertension.

Adjusted for age, gender, race/ethnicity, education level, PIR, health insurance, marital status, alcohol consumption, energy intake, protein intake, carbohydrate intake, fat intake, and fiber intake.

^***^P value < 0.001.

^****^P value < 0.0001.

### 3.6 Dose-response relationship between food intake and LE8 across different live microbe intake groups

**Figure 3:**
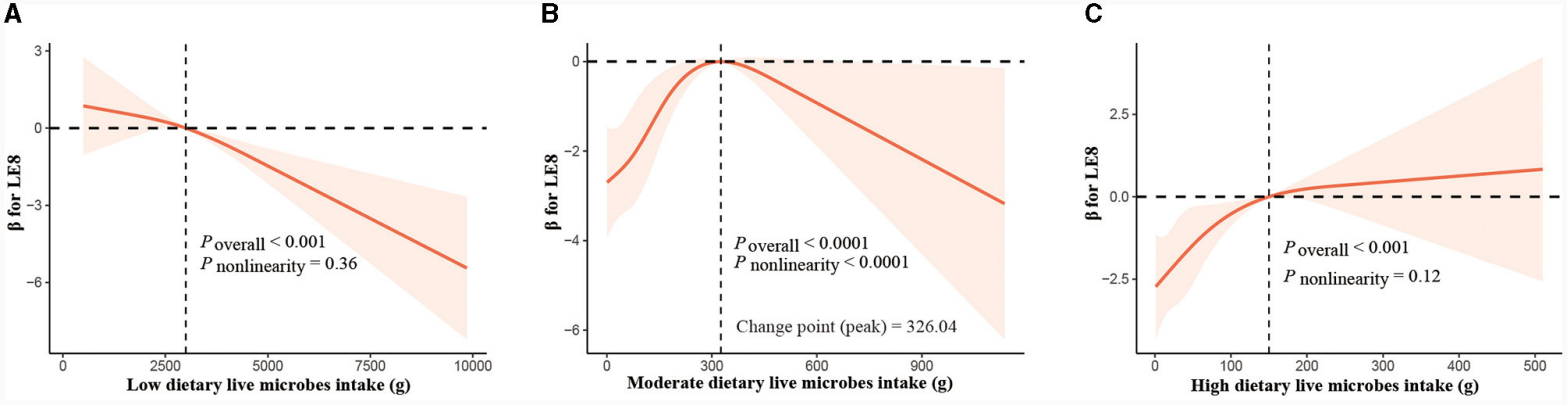
Dose-response relationship curves between total food consumption and adjusted β values for LE8. **(A)** Low live microbes group, **(B)** moderate live microbes group, **(C)** high live microbes group. The model was adjusted for age, gender, race/ethnicity, education level, PIR, health insurance, marital status, alcohol consumption, energy intake, protein intake, carbohydrate intake, fat intake, and fiber intake.

We employed RCS analysis to explore the dose-response relationship between food intake at three different levels of live microbes and CVH. The observations show that in the low live microbe group (Figure 3A), there is a significant negative correlation between the intake of food and LE8 (*P* < 0.001), and there is no significant non-linear trend (non-linear *P* < 0.36). In the medium live microbe group (Figure 3B), the analysis revealed a significant inverted “U” shaped relationship (*P* < 0.0001, non-linear *P* < 0.0001), meaning that the intake of food is positively correlated with LE8 before reaching 326.04 g, and the relationship turns negative after reaching this point. In the high live microbe intake group (Figure 3C), the intake is positively correlated with LE8 (*P* < 0.001), but this correlation is not curvilinear (non-linear *P* = 0.12).

## 4 Discussion

In this nationally representative cross-sectional study, our results demonstrate that the consumption of foods high in live microbes is associated with better CVH. The study finds a significant positive correlation between moderate and high groups of live microbes and LE8, indicating that appropriate consumption of these foods may benefit CVH. Sensitivity analyses further confirm the robustness of this finding, as the positive correlation remains significant even after excluding populations with a history of CVD and other potential confounding factors. Subgroup analyses reveal the universality of this relationship across different populations, although caution should be exercised in generalizing these findings, especially in specific subgroups such as non-Hispanic blacks where the correlation was not significant. Additionally, interaction analyses show that gender differences might influence the relationship between live microbe intake and LE8, suggesting that men and women may respond differently to live microbes consumption. Furthermore, RCS analysis exploring the dose-response relationship between food intake and LE8 at different levels of live microbes reveals a non-linear relationship. Specifically, in the moderate live microbe intake group, there is an inverted “U” shaped relationship between food intake and LE8, implying that a moderate intake of foods with medium levels of live microbes might be more beneficial for CVH. In summary, moderate intake of live microbes is important for maintaining CVH, but its effects may vary among individuals, and personal characteristics should be taken into consideration.

Prior to our study, there had been research exploring the relationship between dietary live microbes and CVD, but the issue was that the diagnosis of CVD was based solely on inquiries about the presence or absence of a relevant medical history, and previous studies focused more on whether CVD occurred (12). Additionally, Macro and colleagues studied dietary live microbes in relation to physiological indicators and found a positive correlation with health, including reductions in triglycerides, systolic blood pressure, and fasting blood sugar levels, as well as an increase in high-density lipoprotein cholesterol levels (14). However, Macro's study focused only on specific indicators and lacked direct linkage in inferring cardiovascular health. Building on these studies, our focus was on the relationship between dietary live microbes and LE8. Since the AHA updated its method for assessing CVH outcomes in 2022, multiple studies have validated the effectiveness of LE8 in predicting CVH and outcomes. Higher levels of LE8 are associated with reduced incidences of coronary heart disease, stroke, and CVD, and are also independently related to lower risks of all-cause and cardiovascular mortality (21, 22). LE8 is a comprehensive indicator, incorporating not only health factors such as blood pressure and lipids but also considering health behaviors like sleep, nicotine exposure, and exercise. In fact, our study also demonstrated significant positive correlations between dietary live microbes and both these aspects. This may help to reveal a deeper connection between live microbes and overall CVH. During the study, we aimed to minimize the confounding effect of dietary factors on the results. Therefore, we utilized the HEI as the scoring standard for diet-related scores in the LE8 evaluation. Compared to the DASH diet score, which emphasizes vegetables, fruits, and low-fat dietary guidelines, HEI is more lenient. Moreover, to further eliminate the confounding effect of diet, we adjusted for calorie intake, protein intake, fat intake, carbohydrate intake, and dietary fiber intake. After these adjustments, medium and high dietary live microbe intake remained associated with higher health behavior and health factor scores. The impact on health behavior scores was still significant, indicating a stable relationship between dietary live microbes intake and higher LE8. Furthermore, we used a variety of analytical methods. To prove the stability of our results, we conducted subgroup analyses, interaction analyses, and sensitivity analyses. Additionally, we used RCS analysis to assess the dose-response relationship between food intake and cardiovascular health. The RCS analysis can accurately capture the complex non-linear relationships between variables and identify key turning points in the dose-response relationship, thereby providing a more precise and scientific basis for risk assessment and dietary guidance.

While the mechanism underlying the relationship between dietary live microbe intake and LE8 scores remains unclear, previous studies have shown that the intake of probiotics or fermented foods can significantly reduce risk factors for cardiac metabolism. Meta-analyses indicate that probiotic supplements can lower blood pressure, blood glucose, total cholesterol, low-density lipoprotein cholesterol, and BMI (19–21). In addition, probiotic supplements can alleviate oxidative stress, maintain gut microbiota homeostasis, and regulate immunity to maintain CVH (22). The metabolic products of gut microbiota, short-chain fatty acids (such as butyrate, propionate, and acetate), can improve gut barrier function, regulate immune and inflammatory responses, and affect the recruitment of immune cells to atherosclerotic plaques, thereby mitigating plaque formation (23–25). In recent years, the gut-brain axis has attracted increasing attention, with gut microbiota and their metabolites playing a crucial role. Current research suggests that gut microbiota and their metabolites can regulate the autonomic nervous system, endocrine system, and immune system, influence the release of neurotransmitters, and affect the activity of the central nervous system, thereby influencing sleep (26, 27). Furthermore, probiotic supplements can enhance physical performance.

To the best of our knowledge, this study is the first to explore the relationship between dietary live microbe and the LE8 in a large US population. The results indicate that consumption of foods providing more dietary live microbes is positively correlated with CVH, potentially increasing scores for healthy behaviors and health factors. Our study has several strengths: firstly, the data used in this study comes from NHANES, and the data we employed underwent rigorous quality control, ultimately including 10,531 participants, which ensures a sizable sample and lends a degree of credibility to the results. Secondly, during the analysis, we considered the differences in daily energy, protein, carbohydrate, and fiber intake among participants with different dietary live microbe groups and adjusted for these factors in the fully adjusted mode. Notably, the influence of dietary live microbes intake on the LE8 remains robust after adjustment. Finally, we used a variety of statistical analysis methods, which helped in identifying special populations and understanding more complex relationships. However, there are certain limitations to the current study. Firstly, this is a cross-sectional study, and therefore, we cannot infer causal relationships. Secondly, we used 24-h dietary recalls to estimate participants' daily dietary intake, which may not accurately reflect their true dietary habits. Thirdly, the content of live microbes in foods was determined through expert literature review and discussion and was not precisely measured, which may affect the results. Fourthly, our conclusions are limited to the US population and may not apply to other regions. Lastly, there may be residual confounding in the study results. Even though we considered as many confounding variables as possible, such as diet and certain diseases, some confounding factors may still affect the results.

## 5 Conclusion

In conclusion, our study indicates that consumption of foods providing more dietary live microbes is positively correlated with scores for healthy behaviors, health factors, and the LE8. This result remains consistent across different populations and is independent of any previous history of cardiovascular-related diseases. More research is needed, particularly using experimental testing methods to determine the specific content of live microbes in various foods. Additionally, to further investigate causality, randomized controlled trials are required.

## Data availability statement

The original contributions presented in the study are included in the article/Supplementary material, further inquiries can be directed to the corresponding author.

## Ethics statement

Ethical approval was not required for the study involving humans in accordance with the local legislation and institutional requirements. Written informed consent to participate in this study was not required from the participants or the participants' legal guardians/next of kin in accordance with the national legislation and the institutional requirements.

## Author contributions

LW: Data curation, Formal analysis, Methodology, Writing – review & editing. SW: Visualization, Writing – original draft. YW: Writing – review & editing. SZ: Writing – review & editing. ZL: Writing – review & editing. YJ: Supervision, Writing – review & editing. XL: Conceptualization, Funding acquisition, Supervision, Writing – review & editing.

## Supplementary material

Supplementary Figure 1 Subgroup Analysis for the associations of Different Dietary Live Microbe Intake and HCVHR. The model was adjusted for age, gender, race/ethnicity, education level, PIR, health insurance, marital status, alcohol consumption, energy intake, protein intake, carbohydrate intake, fat intake, and fiber intake when they were not the strata variables. ^*^*P* < 0.05; ^**^*P* < 0.01; ^***^*P* < 0.001.

Supplementary Table 1 Definition and scoring approach for the American Heart Association's Life's Essential 8 score.

Supplementary Table 2 Healthy Eating Index-2015 Components & Scoring Standards1.

Supplementary Table 3 Definition criteria for variables.

Supplementary Table 4 The clinical characteristics of the study population based on different CVH classifications.

Supplementary Table 5 Association between different dietary live microbe group and health behaviors and health factors.
