# Supplementary figures and images for: Association between dietary live microbe intake and Life's Essential 8 in US adults: a cross-sectional study of NHANES 2005–2018
Source: Front Nutr. 2024 Feb 29;11:1340028. doi: 10.3389/fnut.2024.1340028 (PMC10937585; doi:10.3389/fnut.2024.1340028)

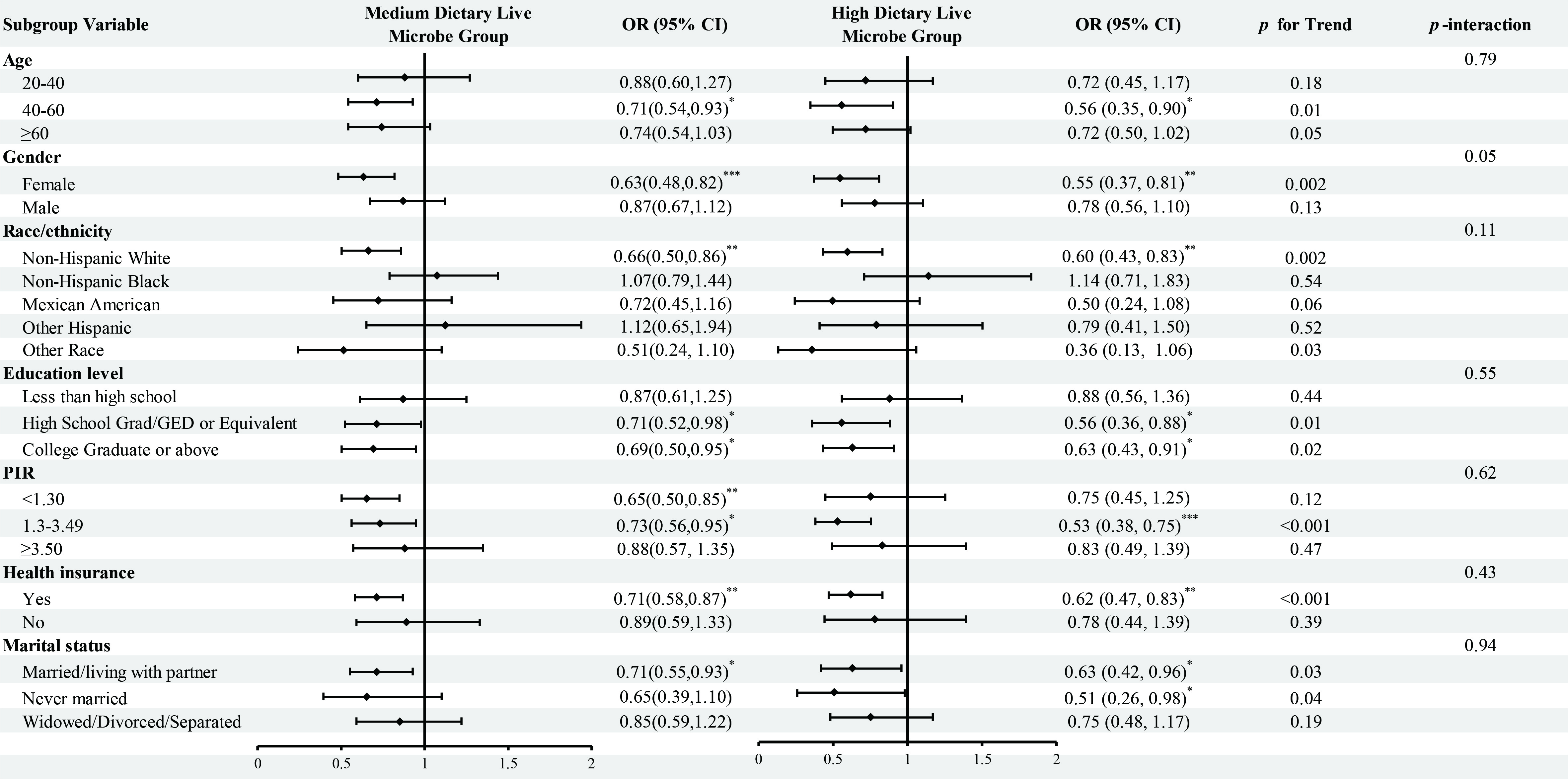

Supplement: Supplementary Figure 1 — Subgroup Analysis for the associations of Different Dietary Live Microbe Intake and HCVHR. The model was adjusted for age, gender, race/ethnicity, education level, PIR, health insurance, marital status, alcohol consumption, energy intake, protein intake, carbohydrate intake, fat intake, and fiber intake when they were not the strata variables. *P < 0.05; **P < 0.01; ***P < 0.001. [file Image_1.jpg]
