# Supplementary material for: Association between dietary live microbe intake and Life's Essential 8 in US adults: a cross-sectional study of NHANES 2005–2018
Source: Front Nutr. 2024 Feb 29;11:1340028. doi: 10.3389/fnut.2024.1340028 (PMC10937585; doi:10.3389/fnut.2024.1340028)
Supplement: Supplementary Table 1 — Definition and scoring approach for the American Heart Association's Life's Essential 8 score. [file Table_1.DOCX]

**Supplementary Table 1**

**Definition and scoring approach for the American Heart Association’s Life’s Essential 8 score.**

| Domain | CVH Metric | Measurement | Quantification and Scoring of CVH Metric |
| --- | --- | --- | --- |
| Health Behaviors | Diet | Healthy Eating Index-2015 diet score percentile | Quantiles of DASH-style diet adherence  Scoring (Population):  Points Quantile  100 ≥95th percentile (top/ideal diet)  80 75th – 94th percentile  50 50th – 74th percentile  25 25th – 49th percentile  0 1st – 24th percentile (bottom/least ideal quartile) |
|  | Physical activity | Self-reported minutes of moderate or vigorous physical activity per week | Metric: Minutes of moderate (or greater) intensity activity per week  Scoring:  Points Minutes  100 ≥150  90 120 – 149  80 90 – 119  60 60 – 89  40 30 – 59  20 1 – 29  0 0 |
|  | Nicotine exposure | Self-reported use of cigarettes or inhaled nicotine- delivery system | Metric: Combustible tobacco use and/or inhaled NDS use; or secondhand smoke exposure  Scoring:  Points Status  100 Never smoker  75 Former smoker, quit ≥5 yrs  50 Former smoker, quit 1 - <5 yrs  25 Former smoker, quit <1 year, or currently using inhaled NDS  0 Current smoker  Subtract 20 points (unless score is 0) for living with active indoor smoker in home |
|  | Sleep health | Self-reported average hours of sleep per night | Metric: Average hours of sleep per night  Scoring:  Points Level  100 7 – <9  90 9 – <10  70 6 – <7  40 5 – <6 or ≥10  20 4 – <5  0 <4 |
| Health Factors | Body mass index | Body weight (kg) divided by height squared (m2) | Metric: Body mass index (kg/m2)  Scoring: Points Level 100 <25  70 25.0 – 29.9  30 30.0 – 34.9  15 35.0 – 39.9  0 ≥40.0 |
|  | Blood lipids | Plasma total and HDL-cholesterol with calculation of non-HDL-cholesterol | Metric: Non-HDL-cholesterol (mg/dL)  Scoring:  Points Level  100 <130  60 130 – 159  40 160 – 189  20 190 – 219  0 ≥220  If drug-treated level, subtract 20 points |
|  | Blood glucose | Fasting blood glucose or casual hemoglobin A1c | Metric: Fasting blood glucose (mg/dL) or Hemoglobin A1c (%)  Scoring:  Points Level  100 No history of diabetes and FBG <100 (or HbA1c < 5.7)  60 No diabetes and FBG 100 – 125 (or HbA1c 5.7-6.4) (Pre-diabetes)  40 Diabetes with HbA1c <7.0  30 Diabetes with HbA1c 7.0 – 7.9  20 Diabetes with HbA1c 8.0 – 8.9  10 Diabetes with Hb A1c 9.0 – 9.9  0 Diabetes with HbA1c ≥10.0 |
|  | Blood pressure | Appropriately measured systolic and diastolic blood pressure | Metric: Systolic and diastolic blood pressure (mm Hg)  Scoring:  Points Level  100 <120/<80 (Optimal)  75 120-129/<80 (Elevated)  50 130-139 or 80-89 (Stage I HTN)  25 140-159 or 90-99  0 ≥160 or ≥100  Subtract 20 points if treated level |

**Reference**

Lloyd-Jones DM, Allen NB, Anderson CAM, et al. Life's Essential 8: Updating and Enhancing the American Heart Association's Construct of Cardiovascular Health: A Presidential Advisory From the American Heart Association. Circulation. Aug 2 2022;146(5):e18-e43.

**Supplementary Table 2**

**Healthy Eating Index-2015 Components & Scoring Standards1**

| Component | Maximum points | Standard for maximum score | Standard for minimum score of zero |
| --- | --- | --- | --- |
| Adequacy | | | |
| Total Fruits[2](https://epi.grants.cancer.gov/hei/developing.html#f2) | 5 | ≥0.8 cup equiv. per 1,000 kcal | No Fruit |
| Whole Fruits[3](https://epi.grants.cancer.gov/hei/developing.html#f3) | 5 | ≥0.4 cup equiv. per 1,000 kcal | No Whole Fruit |
| Total Vegetables[4](https://epi.grants.cancer.gov/hei/developing.html#f4) | 5 | ≥1.1 cup equiv. per 1,000 kcal | No Vegetables |
| Greens and Beans[4](https://epi.grants.cancer.gov/hei/developing.html#f4) | 5 | ≥0.2 cup equiv. per 1,000 kcal | No Dark Green Vegetables or Legumes |
| Whole Grains | 10 | ≥1.5 oz equiv. per 1,000 kcal | No Whole Grains |
| Dairy[5](https://epi.grants.cancer.gov/hei/developing.html#f5) | 10 | ≥1.3 cup equiv. per 1,000 kcal | No Dairy |
| Total Protein Foods[6](https://epi.grants.cancer.gov/hei/developing.html#f6) | 5 | ≥2.5 oz equiv. per 1,000 kcal | No Protein Foods |
| Seafood and Plant Proteins[6](https://epi.grants.cancer.gov/hei/developing.html#f6),[7](https://epi.grants.cancer.gov/hei/developing.html#f7) | 5 | ≥0.8 oz equiv. per 1,000 kcal | No Seafood or Plant Proteins |
| Fatty Acids[8](https://epi.grants.cancer.gov/hei/developing.html#f8) | 10 | (PUFAs + MUFAs)/SFAs ≥2.5 | (PUFAs + MUFAs)/SFAs ≤1.2 |
| Moderation | | | |
| Refined Grains | 10 | ≤1.8 oz equiv. per 1,000 kcal | ≥4.3 oz equiv. per 1,000 kcal |
| Sodium | 10 | ≤1.1 gram per 1,000 kcal | ≥2.0 grams per 1,000 kcal |
| Added Sugars | 10 | ≤6.5% of energy | ≥26% of energy |
| Saturated Fats | 10 | ≤8% of energy | ≥16% of energy |

(1) Intakes between the minimum and maximum standards are scored proportionately.

(2) Includes 100% fruit juice.

(3) Includes all forms except juice.

(4) Includes legumes (beans and peas).

(5) Includes all milk products, such as fluid milk, yogurt, and cheese, and fortified soy beverages.

(6) Includes legumes (beans and peas).

(7) Includes seafood, nuts, seeds, soy products (other than beverages), and legumes (beans and peas).

(8) Ratio of poly- and monounsaturated fatty acids (PUFAs and MUFAs) to saturated fatty acids (SFAs).

Adequacy components represent the food groups, subgroups, and dietary elements that are encouraged. For these components, higher scores reflect higher intakes, because higher intakes are desirable.

Moderation components represent the food groups and dietary elements for which there are recommended limits to consumption. For moderation components, higher scores reflect lower intakes, because lower intakes are more desirable.

**Reference**

1. Krebs-Smith SM, Pannucci TE, Subar AF, et al. Update of the Healthy Eating Index: HEI-2015. J Acad Nutr Diet. Sep 2018;118(9):1591-1602

2. National Cancer Institute. HEI Scoring Algorithm. Accessed August, 2022. https://epi.grants.cancer.gov/hei/hei-scoring-method.html

3. Zhang Y, Sun M, Wang Y, et al. Association of cardiovascular health using Life's Essential 8 with noncommunicable disease multimorbidity [published online ahead of print, 2023 Jul 4]. Prev Med. 2023;174:107607. doi:10.1016/j.ypmed.2023.107607

**Supplement Table 3**

**Definition criteria for variables**

| **Variables** | **Classification** | **Definition** |
| --- | --- | --- |
| Alcohol consumption | Never | Drink less than 12 times in lifetime. |
|  | Former | Did not drink last year, but had more than 12 drinks in a year, or did not drink last year but had more than 12 drinks in a lifetime. |
|  | Mild | For female, consuming up to 1 alcoholic drink per day, and for male, consuming up to 2 alcoholic drinks per day. |
|  | Moderate | 1. For female consume 2 alcoholic drinks per day, and for male consume 3 alcoholic drinks per day.  2. Binge drinking of 2 or more times a month but less than 5 times (both males and females). |
|  | Heavy | 1. For female consume 3 alcoholic drinks or more per day, and for male consume 4 alcoholic drinks or more per day. 2. Binge drinking of 5 or more times a month (both males and females). |
| Obesity status | Normal weight | BMI < 25 kg/m^2^ |
|  | Overweight | BMI ≥ 25 kg/m^2^ and BMI < 30 kg/m^2^ |
|  | Obesity | BMI ≥ 30 kg/m^2^ |
| Cardiovascular diseases | Yes | Having a history of any of the following conditions: coronary heart disease, congestive heart failure, angina, heart attack, and stroke. |
|  | No | None of the above. |
| Diabetes mellitus | Yes | 1. Doctor told you have diabetes,  2. Glycohemoglobin (HbA1c) ≥ 6.5%,  3. Fasting glucose ≥ 7.0 mmol/l,  4. Random blood glucose ≥ 11.1 mmol/l,  5. Two-hour OGTT blood glucose ≥ 11.1 mmol/l,  6. Use of diabetes medication or insulin. |
|  | No | None of the above. |
| Hypertension | Yes | 1. Doctor told you hypertension,  2. Use blood pressure medications,  3. Average systolic blood pressure ≥140, or diastolic blood pressure ≥ 90 (Take the average of three blood pressure readings) |
|  | No | None of the above. |
| Hyperlipidemia | Yes | 1. Triglycerides (TG) ≥ 150mg/dl,  2. Total cholesterol (TC ) ≥ 130mg/dl,  3. Low density lipoprotein (LDL) < 40 mg/dl for males or < 50 mg/dl for females,  4. High density lipoprotein (HDL) < 40 mg/dl for males or < 50 mg/dl for females,  5. Use lipid-lowering medication. |
|  | No | None of the above. |

**Supplement Table 4**

**The clinical characteristics of the study population based on different CVH classifications.**

| **Characteristic** | **Total (n=10531)** | **Low CVH**^†^  **(n=2170)** | **Moderate CVH**  **(n=1242)** | **High CVH**  **(n=7119)** | ***p*-Value** |
| --- | --- | --- | --- | --- | --- |
| **Age [years]** | 47.59±0.30 | 53.80±0.60 | 48.78±0.31 | 41.91±0.46 | < 0.0001 |
| **Age group(%)** |  |  |  |  | < 0.0001 |
| 20-40 | 3379(35.49) | 179(17.16) | 2077(32.70) | 1123(50.24) |  |
| 40-60 | 3567(38.36) | 489(45.90) | 2413(39.06) | 665(33.52) |  |
| ≥60 | 3585(26.15) | 577(36.94) | 2590(28.25) | 418(16.25) |  |
| **Gender (%)** |  |  |  |  | < 0.0001 |
| Female | 5413(51.80) | 652(54.42) | 3434(48.58) | 1327(59.60) |  |
| Male | 5118(48.20) | 593(45.58) | 3646(51.42) | 879(40.40) |  |
| **Race/ethnicity (%)** |  |  |  |  | < 0.0001 |
| Non-Hispanic White | 4971(71.16) | 555(67.01) | 3350(71.06) | 1066(73.04) |  |
| Non-Hispanic Black | 2052(10.28) | 361(17.36) | 1439(10.87) | 252(5.89) |  |
| Mexican American | 1547(7.36) | 158(6.96) | 1074(7.45) | 315(7.30) |  |
| Other Hispanic | 959(4.78) | 114(4.64) | 635(4.76) | 210(4.88) |  |
| Other Race | 1002(6.42) | 57(4.03) | 582(5.86) | 363(8.89) |  |
| **Education level (%)** |  |  |  |  | < 0.0001 |
| Less than high school | 2255(13.97) | 414(24.97) | 1595(15.29) | 246(6.08) |  |
| High School Grad/GED or Equivalent | 2401(22.80) | 372(34.65) | 1732(24.87) | 297(12.53) |  |
| College Graduate or above | 5875(63.23) | 459(40.38) | 3753(59.84) | 1663(81.40) |  |
| **PIR (%)** |  |  |  |  | < 0.0001 |
| <1.30 of FPL | 3031(19.21) | 514(31.77) | 2053(19.47) | 464(13.63) |  |
| 1.3-3.49 of FPL | 4067(36.48) | 529(44.24) | 2782(37.71) | 756(30.12) |  |
| ≥3.50 of FPL | 3433(44.31) | 202(23.99) | 2245(42.83) | 986(56.25) |  |
| **Health insurance** |  |  |  |  | < 0.001 |
| Yes | 8447(84.00) | 997(80.87) | 5647(83.25) | 1803(87.26) |  |
| No | 2084(16.00) | 2084(16.00) | 248(19.13) | 1433(16.75) |  |
| **Marital status (%)** |  |  |  |  | < 0.0001 |
| Married/living with partner | 6444(64.98) | 691(59.49) | 4361(65.06) | 1392(66.91) |  |
| Never married | 1828(17.38) | 169(13.99) | 1112(15.74) | 547(23.16) |  |
| Widowed/Divorced/Separated | 2259(17.64) | 385(26.52) | 1607(19.20) | 267(9.93) |  |
| **Alcohol consumption (%)** |  |  |  |  | < 0.0001 |
| Never | 1341(10.20) | 143(9.43) | 875(9.82) | 323(11.55) |  |
| Former | 1751(13.39) | 345(25.27) | 1213(14.05) | 193(7.01) |  |
| Mild | 3796(38.76) | 344(28.06) | 2516(37.84) | 936(45.42) |  |
| Moderate | 1636(17.84) | 152(15.25) | 1074(17.19) | 410(20.65) |  |
| Heavy | 2007(19.80) | 261(22.00) | 1402(21.11) | 344(15.38) |  |
| **LE8** | 69.03±0.29 | 42.32±0.23 | 66.31±0.14 | 86.85±0.15 | < 0.0001 |
| **Energy intake [kcal/day]** | 2101.73±10.14 | 2027.93±34.74 | 2117.53±13.29 | 2087.13±20.83 | 0.002 |
| **Protein intake [g/day]** | 82.77±0.44 | 77.88±1.56 | 82.84±0.60 | 84.48±0.97 | < 0.001 |
| **Carbohydrate intake [g/day]** | 250.22±1.37 | 242.65±4.23 | 250.52±1.68 | 252.35±2.78 | < 0.001 |
| **Fiber intake [g/day]** | 17.04±0.16 | 13.62±0.28 | 16.17±0.16 | 20.77±0.32 | < 0.0001 |
| **Fat intake [g/day]** | 81.33±0.47 | 80.21±1.67 | 82.51±0.63 | 78.54±0.97 | 0.01 |
| **BMI** | 28.96±0.11 | 35.18±0.31 | 29.71±0.09 | 24.51±0.11 | < 0.0001 |
| **Obesity (%)** |  |  |  |  | < 0.0001 |
| Normal weight | 3058(30.41) | 83(6.15) | 1645(22.56) | 1330(61.29) |  |
| Over weight | 3487(33.05) | 234(17.11) | 2533(35.53) | 720(32.45) |  |
| Obesity | 3986(36.54) | 928(76.74) | 2902(41.90) | 156(6.26) |  |
| **CVD (%)** |  |  |  |  | < 0.0001 |
| Yes | 1163(8.81) | 323(22.83) | 758(8.93) | 82(3.02) |  |
| No | 9368(91.19) | 922(77.17) | 6322(91.07) | 2124(96.98) |  |
| **DM (%)** |  |  |  |  | < 0.0001 |
| Yes | 1980(13.87) | 589(40.82) | 1343(14.50) | 48(1.69) |  |
| No | 8551(86.13) | 656(59.18) | 5737(85.50) | 2158(98.31) |  |
| **Hypertension (%)** |  |  |  |  | < 0.0001 |
| Yes | 4472(37.44) | 921(70.70) | 3249(42.21) | 302(11.49) |  |
| No | 6059(62.56) | 324(29.30) | 3831(57.79) | 1904(88.51) |  |
| **Hyperlipidemia (%)** |  |  |  |  | < 0.0001 |
| Yes | 7583(70.53) | 1141(92.29) | 5398(76.41) | 1044(46.01) |  |
| No | 2948(29.47) | 104(7.71) | 1682(23.59) | 1162(53.99) |  |
| **Dietary live microbes group (%)** |  |  |  |  | < 0.0001 |
| Low | 3719(31.83) | 593(45.73) | 2614(34.17) | 512(20.03) |  |
| Medium | 4339(39.42) | 456(34.84) | 2926(39.44) | 957(41.14) |  |
| High | 2473(28.75) | 196(19.42) | 1540(26.39) | 737(38.83) |  |
| **HCVHR**^‡^ **(%)** |  |  |  |  | < 0.0001 |
| Yes | 1245(9.41) | 1245(100.00) | 0(0.00) | 0(0.00) |  |
| No | 9286(90.59) | 0(0.00) | 7080(100.00) | 2206(100.00) |  |
| **Year Cycle (%)** |  |  |  |  | 0.02 |
| 2005-2010 | 4722(42.75) | 578(43.43) | 3259(44.00) | 885(39.06) |  |
| 2010-2018 | 5809(57.25) | 667(56.57) | 3821(56.00) | 1321(60.94) |  |

CVH, cardiovascular health; BMI, body mass index; LE8, life’s essential 8; HEI, healthy eating index; SBP, systolic blood pressure; DBP, diastolic blood pressure; PIR, Ratio of family income to poverty; CVD, Cardiovascular disease; DM, Diabetes Mellitus; HCVHR, high cardiovascular health risk

^†^ Low CVH was defined as a LE8 score of 0-49, moderate CVH of 50-79, and high CVH of 80-100

^‡^ HCVHR was defined as participants with LE8 scores less than 50.

**Supplementary Table 5.**

**Association between different dietary live microbe group and health behaviors and health factors.**

| **Outcome** | **Model** | **Low Dietary Live Microbe Group**  **β（95%CI）** | **Medium Dietary Live Microbe Group**  **β（95%CI）** | **High Dietary Live Microbe Group**  **β（95%CI）** | ***p* for Trend** |
| --- | --- | --- | --- | --- | --- |
| health behaviors | Crude | 1.00 (Reference) | 8.60(7.51-9.69)^****^ | 10.42(9.13-11.71)^****^ | <0.0001 |
|  | Model 1 | 1.00 (Reference) | 7.20(6.17-8.24)^****^ | 8.14(6.90-9.37)^****^ | <0.0001 |
|  | Model 2 | 1.00 (Reference) | 6.33(5.33-7.34)^****^ | 7.02(5.82-8.23)^****^ | <0.0001 |
|  | Model 3 | 1.00 (Reference) | 4.35(3.41-5.30)^****^ | 5.12(3.95-6.28)^****^ | <0.0001 |
| health factors | Crude | 1.00 (Reference) | 1.09(-0.16-2.35) | 3.53(2.38-4.69)^****^ | <0.0001 |
|  | Model 1 | 1.00 (Reference) | 2.13(1.04-3.22)^***^ | 3.09(1.94-4.23)^****^ | <0.0001 |
|  | Model 2 | 1.00 (Reference) | 1.95(0.88-3.01)^***^ | 2.81(1.72-3.90)^****^ | <0.0001 |
|  | Model 3 | 1.00 (Reference) | 1.64(0.57-2.71)^**^ | 2.76(1.69-3.84)^****^ | <0.0001 |

Model 1: Adjusted for age, gender, race/ethnicity, and education level.

Model 2: Further adjusted for PIR, health insurance, marital status, and alcohol.

Model 3: Further adjusted for energy intake, protein intake, carbohydrate intake, fat intake, and fiber intake.

* p value < 0.05, ** p value < 0.01, *** p value < 0.001, **** p value < 0.0001.
